# Development of a core outcome set for use in adult primary glioma phase III interventional trials: A mixed methods study

**DOI:** 10.1093/noajnl/vdad096

**Published:** 2023-08-02

**Authors:** Ameeta Retzer, Elin Baddeley, Stephanie Sivell, Hannah Scott, Annmarie Nelson, Helen Bulbeck, Kathy Seddon, Robin Grant, Richard Adams, Colin Watts, Olalekan Lee Aiyegbusi, Pamela Kearns, Samantha Cruz Rivera, Linda Dirven, Melanie Calvert, Anthony Byrne

**Affiliations:** Centre for Patient Reported Outcomes Research (CPROR), Institute of Applied Health Research, University of Birmingham, Birmingham, UK; National Institute for Health Research (NIHR) Applied Research Collaboration West Midlands (ARC WM), Birmingham, UK; NIHR Birmingham Biomedical Research Centre (BRC), University of Birmingham, Birmingham, UK; Marie Curie Palliative Care Research Centre, Division of Population Medicine, Cardiff University School of Medicine, Cardiff, UK; Marie Curie Palliative Care Research Centre, Division of Population Medicine, Cardiff University School of Medicine, Cardiff, UK; Division of Research and Evaluation, Office for Standards in Education, Childrens' Services and Skills (OFSTED), Bristol, UK; Marie Curie Palliative Care Research Centre, Division of Population Medicine, Cardiff University School of Medicine, Cardiff, UK; Brainstrust - The Brain Cancer People, UK; Cardiff University, Cardiff, UK; Department of Clinical Neurosciences, Royal Infirmary of Edinburgh, Edinburgh, UK; Centre for Trials Research, Cardiff University, Cardiff, UK; Institute of Cancer and Genomic Sciences, University of Birmingham, UK; Centre for Patient Reported Outcomes Research (CPROR), Institute of Applied Health Research, University of Birmingham, Birmingham, UK; National Institute for Health Research (NIHR) Applied Research Collaboration West Midlands (ARC WM), Birmingham, UK; Birmingham Health Partners Centre for Regulatory Science and Innovation, University of Birmingham, Birmingham, UK; NIHR Birmingham Biomedical Research Centre (BRC), University of Birmingham, Birmingham, UK; NIHR Birmingham-Oxford Blood and Transplant Research Unit (BTRU) in Precision Transplant and Cellular Therapeutics, University of Birmingham, Birmingham, UK; Institute of Cancer and Genomic Sciences, University of Birmingham , UK; NIHR Birmingham Biomedical Research Centre (BRC), University of Birmingham, Birmingham, UK; Cancer Research UK Clinical Trials Unit, Institute of Cancer and Genomic Sciences, University of Birmingham , UK; Centre for Patient Reported Outcomes Research (CPROR), Institute of Applied Health Research, University of Birmingham, Birmingham, UK; Birmingham Health Partners Centre for Regulatory Science and Innovation, University of Birmingham, Birmingham, UK; Department of Neurology, Leiden University Medical Center, Leiden, The Netherlands; Department of Neurology, Haaglanden Medical Center, The Hague, The Netherlands; Centre for Patient Reported Outcomes Research (CPROR), Institute of Applied Health Research, University of Birmingham, Birmingham, UK; National Institute for Health Research (NIHR) Applied Research Collaboration West Midlands (ARC WM), Birmingham, UK; Birmingham Health Partners Centre for Regulatory Science and Innovation, University of Birmingham, Birmingham, UK; NIHR Birmingham Biomedical Research Centre (BRC), University of Birmingham, Birmingham, UK; Midlands Health Data Research UK, Birmingham, UK; NIHR Birmingham-Oxford Blood and Transplant Research Unit (BTRU) in Precision Transplant and Cellular Therapeutics, University of Birmingham, Birmingham, UK; Marie Curie Palliative Care Research Centre, Division of Population Medicine, Cardiff University School of Medicine, Cardiff, UK

**Keywords:** Delphi, neuro-oncology, outcomes, primary glioma, trials

## Abstract

**Background:**

Glioma interventional studies should collect data aligned with patient priorities, enabling treatment benefit assessment and informed decision-making. This requires effective data synthesis and meta-analyses, underpinned by consistent trial outcome measurement, analysis, and reporting. Development of a core outcome set (COS) may contribute to a solution.

**Methods:**

A 5-stage process was used to develop a COS for glioma trials from the UK perspective. Outcome lists were generated in stages 1: a trial registry review and systematic review of qualitative studies and 2: interviews with glioma patients and caregivers. In stage 3, the outcome lists were de-duplicated with accessible terminology, in stage 4 outcomes were rated via a 2-round Delphi process, and stage 5 comprised a consensus meeting to finalize the COS. Patient-reportable COS outcomes were identified.

**Results:**

In Delphi round 1, 96 participants rated 35 outcomes identified in stages 1 and 2, to which a further 10 were added. Participants (77/96) rated the resulting 45 outcomes in round 2. Of these, 22 outcomes met a priori threshold for inclusion in the COS. After further review, a COS consisting of 19 outcomes grouped into 7 outcome domains (survival, adverse events, activities of daily living, health-related quality of life, seizure activity, cognitive function, and physical function) was finalized by 13 participants at the consensus meeting.

**Conclusions:**

A COS for glioma trials was developed, comprising 7 outcome domains. Additional research will identify appropriate measurement tools and further validate this COS.

**Key Points**

- This manuscript describes the development of a core outcome set for use in interventional trials in adult glioma.
- The core outcome set relates to all outcome types and applies across the spectrum of adult glioma.
- Outcomes that are patient reportable are identified.

**Importance of Study**

Glioma patients’ potentially poor prognosis and high-symptom burden has led to greater emphasis on their quality of survival, maintaining neurocognitive and physical function, and overall health-related quality of life throughout the disease trajectory. Therefore, glioma intervention studies should collect a range of data aligned with patient priorities to enable assessment of net clinical treatment benefit. When assessing interventions, patient-reported outcomes allow insight into how treatment affects patients’ perceived functioning, complementing other outcome data such as survival and radiological response, aiding physicians and patients in clinical decision-making. To this end, ascertaining a core set of relevant outcomes is of utmost importance but is currently lacking for glioma trials. Standardizing analysis, interpretation, and reporting of outcomes is necessary to optimize evidence synthesis. Core outcome set development is an important first step.

Gliomas account for 27% of all brain tumors, and for 80% of malignant brain tumors.^1,2^ They are a heterognous group of cancers with variable prognosis, graded from least to most aggressive (1 to 4).^3^ Brain tumor classification has evolved following developments in molecular diagnostics accounting for the diversity of glioma,^4^ reflecting different survival rates. Presence of a tumor and its treatment have negative effects on patients’ functioning and well-being and those close to them, causing disease-specific symptoms (eg, neurocognitive deficits, motor dysfunction, seizures), in addition to general cancer symptoms (eg, fatigue and pain).

The poor prognosis and high-symptom burden of glioma patients has led to a growing awareness of their quality of survival^5^—a patient-centric concept describing how “well’ a person survives. In cases of modest survival benefits, maintaining neurocognitive and physical function and other aspects of health-related quality of life (HRQOL) are critical.^6^ Thus, glioma interventional studies should collect data from the patient perspective that enables assessment of net treatment benefit. This stance is echoed by regulators with an expanded treatment tolerability definition to better measure patient experience.^7^ The European Medicines Agency (EMA) supports using patient-reported outcome (PRO) data to assess efficacy and tolerability during cancer product approval,^8^ and the European Union’s Innovative Medicines Initiative are developing an international ecosystem to incorporate PROs^9^ and fund efforts to standardize use, analysis, and interpretation of PRO data in cancer clinical trials^10^; the UK Medical and Healthcare Products Regulatory Agency (MHRA) includes PRO data in its Innovative Licensing and Access Pathway^11^; and the 21st Century Cares Act directs the US Food and Drug Administration (FDA) to report on use of patient experience data in its decision-making.^12^

Interpreting net treatment benefits of interventions requires effective data synthesis and meta-analyses, which are dependent on consistent use of outcomes across trials. However, a standard categorical system for outcomes is lacking in cancer clinical trials generally^13^ and brain tumors specifically^14^ where selective outcome reporting, including of PROs, is common. This introduces risks of bias and research waste, hindering evidence synthesis. Core outcome sets (COS) have developed to determine and describe the minimum outcomes to be collected and reported in all clinical trials of a specific condition,^15^ acquired through systematic processes and consensus-generation involving diverse stakeholders. COS facilitate consistent outcome collection and reporting and, alongside other standardization activity, can promote data synthesis and meta-analyses, reduce research waste and inform patient-centered care.^15^

The FDA recommends core PRO data collection in cancer trials relating to disease-related symptoms, symptomatic adverse events, overall side-effects, physical function, and role function.^16^ For high-grade glioma, patient-reported core symptom and function constructs have also been identified by international working groups involving key stakeholders, which are recommended for use across studies.^14,17^ PROs complement other Clinical Outcome Assessments (COAs) including Clinician Reported Outcomes (ClinRO), Observer Reported Outcomes (ObsRO), and Performance Outcomes (PerfO) in describing how a patient feels and functions,^18^ particularly when ensuring complete symptomatic reporting.^19^ How the collection of PROs compliments other COAs such as survival and radiological response in the context of glioma is unclear.

This paper describes development of a COS for all gliomas, inclusive of all types of outcome data, for use in adult primary glioma phase III interventional trials (including systemic anticancer treatments such as immunotherapy and chemotherapy, radiotherapy, surgery, and supportive care. Due to interest in core PROs in cancer, our secondary aim is to identify which COS outcomes can be patient-reported. Further methodological detail and rationale are published elsewhere.^20^

## Methods

### Ethics and Transparency Statement

Ethical approval was granted by Cardiff University School of Medicine Research Ethics Committee (REF: SMREC 21/59). The research was registered with the COMET (Core Outcome Measures in Effectiveness Trials) Initiative and PROSPERO (CRD42021236979). It is reported in adherence with COS-STAR^21^ (Supplementary Table S1) and GRIPP2-SF^22^ (Supplementary Table S2).

### Patient and Public Contribution

Patient focus was maintained through Public and Patient Involvement (PPI) membership of the steering and study management groups. Through separate meetings with the core team, PPI team members advised on recruitment strategies, outcome definitions, qualitative findings, Delphi piloting, and patient-facing documentation. Opportunities to pilot the Delphi survey were offered to the public. A novel PPI tracking tool^23^ licensed at Cardiff University was piloted to plan and document PPI activities according to the National Standards for PPI in Research.^24^ This will be reported in full, separately.

### Research Design

**Figure 1.**
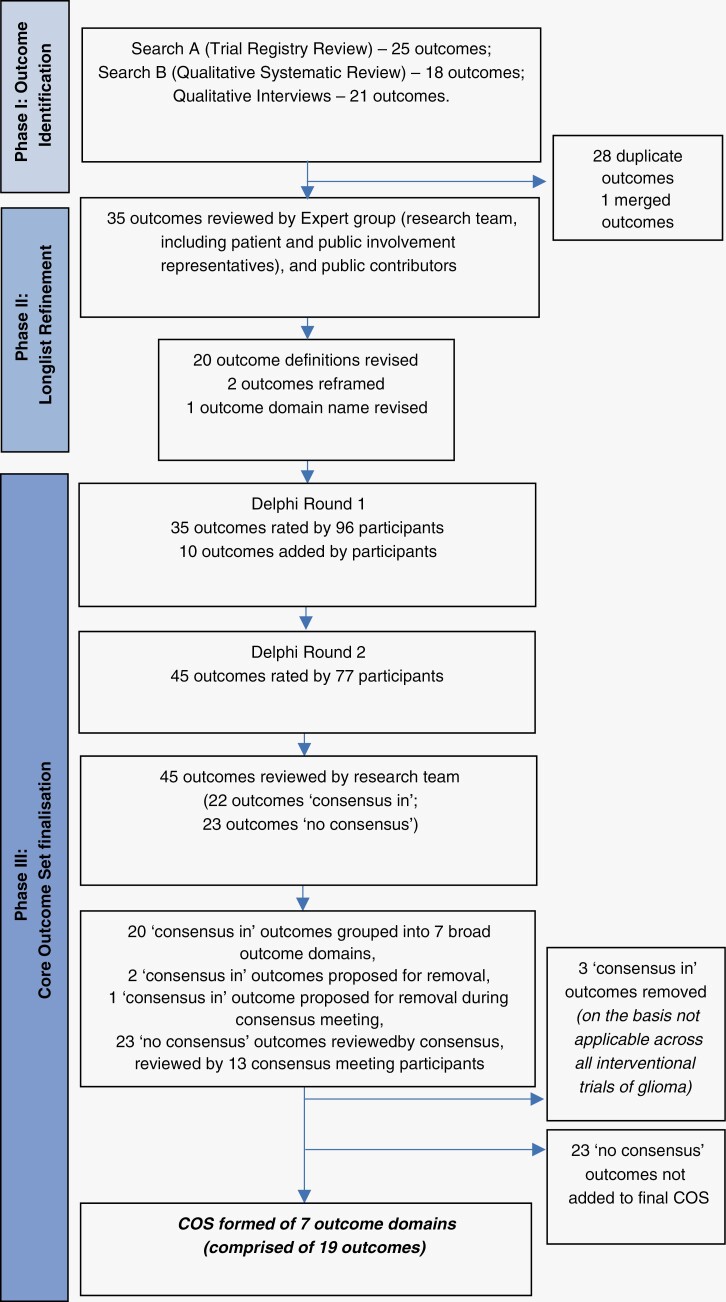
Illustration of core outcome set development process.

We describe the COS development process through a mixed method, 5-stage approach, in accordance with accepted COS methodology (Figure 1). Our methodological approach is further detailed in the study protocol paper.^20^ The study was developed on behalf of a subgroup of the UK National Cancer Research Institute’s Brain Clinical Studies Group with representation from neuro-oncologists, neurologists, allied health professionals, and other neuro-oncology specialities as well as patient and public representatives. The study team included clinicians, qualitative researchers, outcome methodologists, and public contributors.

### Stage 1: Evidence Review

A review of clinical trial registries (Search A) and systematic review of published qualitative literature (Search B).

#### Search A.

Two researchers (A.R./E.B.) independently searched ClinicalTrials.gov^25^ and the ISRCTN Registry^26^ without restriction by date. Results were screened independently and basic trial data were extracted including dates, all primary and secondary outcomes, and outcome measures. Data were cross-referenced with the registration entry for completeness and the protocol when available. The most recent of these were used. The outcomes were deduplicated and formulated into a list.

#### Search B.

MEDLINE, EMBASE, CINAHL, Web of Science, PsycINFO, Cochrane Central Register of Controlled Trials and Cochrane Library were searched between January 1, 2006 and August 9, 2021 (Supplementary Table S3). Key author reference lists and journals were hand searched. Studies containing qualitative data, published in the English language, pertaining to the lived experience of patients with primary glioma by their own account or that of informal caregivers and/or healthcare professionals were included. Two reviewers independently reviewed titles, abstracts, and full text studies (E.B. and S.S.), a third (A.B.) reconciled discrepancies. Methodological quality was assessed by 2 reviewers (E.B., S.S.) and was not a criterion for exclusion.^27^

Two reviewers independently extracted outcomes meeting the predefined outcome definition using a bespoke standardized data collection form. Outcomes were confirmed following discussion with the study team, including PPI team members.

### Stage 2: Semi-Structured Interviews

#### Qualitative sampling, recruitment, and data collection.

Semi-structured interviews were conducted with adults self-reporting histological primary glioma diagnoses (*n* = 19), and adult caregivers in an interview dyad (*n* = 7) where this was preferred by participants, over telephone or video-conferencing software. Interviews were audio recorded and transcribed verbatim. Diagnostic information was recorded using participants’ own terminology and included terms relating to tumor type and grading. Caregivers were informal carers, including relatives or friends, providing the majority of support to the patient and able to estimate the patient’s priorities. Participants were recruited through 2 charities brainstrust,^28^ The Brain Tumour Charity^29^, and social media. Potential participants contacted the research team expressing interest. Recruitment was monitored by the study team to promote diversity in glioma type, actively seeking balance between types. Age, ethnicity, and gender were monitored secondarily, although participants were not selected on this basis. Recruitment ended when no new concepts were obtained from the interviews,^30^ determined through discussion within the research team. In accordance with the Mental Capacity Act (2005),^31^ patient participants were assumed to have capacity unless proven otherwise.

A bespoke semi-structured interview guide (Supplementary Table S4) was used to understand patient experiences of their glioma and its treatment, and the outcomes which they prioritized within that experience, alongside caregiver interpretation of patients’ experiences and priorities within dyads. Participants were also asked directly which outcomes were of most importance to them during treatment, including in clinical trials. Interview guides were piloted by PPI team members and adapted iteratively as interviews progressed as necessary.

#### Analyses.

Transcribed and anonymized interview data were coded using NVivo 12 software^32^ and thematically analyzed. Patient and caregiver data were formulated into separate accounts. A coding framework was developed by 2 research team members (E.B., S.S.) experienced in qualitative methods and reviewed by the study team (A.N., A.R., A.B.) including PPI team members (K.S., H.B.). The coding framework was refined iteratively until thematic analysis^33^ of all transcripts had been completed, generating themes and subthemes.

An outcome was defined as “The variable to be measured… the measurable characteristic that is influenced or affected by an individuals’ baseline state or an intervention as in a clinical trial or other exposure.”^34^ Items meeting this definition were extracted from themes and subthemes (E.B.) and were confirmed following discussion with the study team (A.B., A.R., S.S.). Items from the qualitative data sources not meeting this definition but appearing important to key stakeholders and of relevance to clinical care experiences were recorded and reported separately.^35^

### Stage 3: Outcome List Refinement

Prior to outcome extraction, a broad classification framework was developed^36^ serving as a categorical system to present the outcomes accessibly. Each category represented domains and subdomains broadly measuring particular intervention effects. The Stages 1 and 2 outcomes were independently categorized by 2 researchers. Differences were resolved through discussion.

A deduplicated longlist of outcomes was derived from the Stages 1 and 2 outcome lists. Origins of each outcome and any subsequent decisions were recorded. Outcome definitions were deliberately kept narrow and specific to allow interrogation of each particular concept during their rating by participants in the Delphi survey (Stage 4) and inclusion in the final COS. The study team refined the language used to describe the outcomes by referring to wording in existing outcome measures and with specific input from PPI team members.

### Stage 4: Modified 2-Round Delphi Survey

A modified 2-round Delphi survey was used to assess the relative importance of outcomes in the Stage 3 outcome list, to reach consensus on which should form the COS for glioma trials. The team reviewed the Delphi survey structure, and it was piloted with PPI team members and interview participants wishing to participate further. The survey language and layout were revised based on their feedback to promote accessibility.

#### Recruitment and process.

Participants with professional or personal experience of glioma care, treatment, and research were recruited purposively.^37^ Patients and caregivers were contacted through brainstrust, The Brain Tumour Charity, and social media platforms. Healthcare professionals, researchers, and policy-makers were recruited through NCRI, Tessa Jowell BRAIN MATRIX Trial Platform, clinical trial units, snowballing, and via study group known contacts. In addition, support groups working with specific ethnic groups (Cancer Black Care, Asian Women’s Cancer Group) were contacted. Recruitment was monitored, and efforts were directed as required to promote inclusion of diverse participants representing key stakeholders. Regulatory experts (FDA, MHRA, and EMA) and those working in the pharmaceutical industry, based in the UK and internationally, were contacted through study group known contacts and snowballing. During Delphi registration, participants chose the stakeholder group they most identified with, noting identification with other stakeholder groups besides their primary where applicable.

The Delphi survey was hosted on a bespoke online platform^38^ and could be completed independently online or by telephone with a member of the research team. The Delphi survey presented the Stage 3 outcome list, each in their category. Participants rated each of the outcomes on a 9-point Likert scale, ranging from 1 to 9 indicating not important to very important. Round 1 participants could add outcomes they felt were missing. All original outcomes were presented in round 2. Outcomes added by participants in round 1 were reviewed by the research team and those meeting the criteria for inclusion (applicability to adult primary glioma phase III interventional trials) were presented in round 2. In round 2, participants were shown their own rating for each outcome and those for each stakeholder group, and invited to amend their score if they wished. Participants could share their scoring rationale for each outcome using free text boxes. Content analyses were undertaken on the free-text responses.

The thresholds for inclusion in or exclusion from the COS were confirmed a priori, informed by comparable studies.^36^ Outcomes were proposed for inclusion in the final COS if ≥70% respondents rated the outcome as 7–9 and ≤15% rated the outcome as 1–3. Outcomes were proposed for exclusion from the final COS if ≥70% respondents rated the outcome as 1–3 and ≤15% rated the outcome as 7–9. Outcomes not reaching agreement for inclusion or exclusion after the Delphi rounds were discussed in the consensus meeting, together with the outcomes proposed for inclusion and exclusion. If a large number of outcomes did not reach agreement, to ensure this task was manageable, the study team proposed that only outcomes receiving a rating of 7–9 by ≥60% of respondents were presented to the participants for consideration for inclusion in or exclusion from the COS.

### Stage 5: Consensus Meeting

Outcomes meeting the prespecified criteria for inclusion were reviewed again by the study team to ensure that they were applicable to adult primary glioma phase III interventional trials. Those proposed for removal were presented at the consensus group meeting. The remaining outcomes were grouped into broad outcome domains based on their original categories.

The consensus meeting was held virtually. Purposive sampling was used to promote inclusion of participants with personal experience representing the breadth of glioma types and professional experience capturing key stakeholders, namely healthcare professionals occupying a range of roles in neuro-oncology multidisciplinary teams, researchers, policy-makers, regulators,^36^ and those working in the third sector. Participants were recruited through the sources detailed in earlier stages. In addition, third sector organizations, including support groups working with specific ethnic groups (Cancer Black Care, Asian Women’s Cancer Group), were invited. Recruitment was monitored and efforts were directed as required to promote inclusion of diverse participants representing key stakeholders. The meeting was audiorecorded and notes taken throughout. Decisions made during the consensus meeting were made through anonymous voting using the integrated videoconferencing voting software. Decisions proceeded if ratified by ≥70% of the group; however, when there was <100% consensus, decisions were discussed until those in disagreement were satisfied their views were considered, and the decision could proceed. The final COS was discussed, validated, and confirmed.

#### Identification of patient-reportable outcomes.

Outcomes and outcome measures reported and captured in Search A were cross-referenced with outcomes in the final COS. The measures were checked using the ePROVIDE PROQOLID database^39^ to ascertain if they were patient-reported or composite measures with a patient-reported component. The full measures were retrieved and items cross-referenced with the subdomains in the final COS to identify overlap signifying potential for patient-reporting.

## Results

### Stage 1

#### Search A.

The ISRCTN and clinicaltrials.gov databases were searched on July 30, 2021. A total of 236 records were screened, identifying 91 trials eligible for inclusion (Supplementary Table S5), reported separately.^40^ All outcomes confirmed in the trial descriptions as intended for use were captured across all intervention types and across all glioma trials.

#### Search B.

The database searches were undertaken on August 9, 2021. A total of 8265 records were screened, identifying 21 publications eligible for inclusion (Supplementary Table S6).

The registry review identified 25 outcomes, of which 15 were not identified in the qualitative interviews or systematic review. The qualitative systematic review identified 18 outcomes, which were also identified in the registry review or the semi-structured interviews.

### Stage 2

Interviews (*n* = 19) were held between August 10 2021 and October 29, 2021, involving 19 people with glioma diagnoses and 7 informal caregivers (Table 1). Interviews lasted between 33 minutes and 1 hour 29 minutes.

**Table 1.** Demographic Table Lived Experience Participants—Patients and Carers

Demographics	Patients	Caregivers
Total	19	7
Age (mean, range)	46, 27–66	50, 39–60
Gender		
Female	7	5
Male	12	2
Ethnicity		
White, British	17	6
Asian/Asian British— Indian, Pakistani, Bangladeshi, Chinese, any other Asian background	2	0
Unclear	0	1
Diagnosis	2007–2021	N/A
*Glioma type*		
Glioblastoma	6	3
Astrocytoma	4	1
Oligodendrog lioma	3	1
Anaplastic astrocytoma	2	0
Anaplastic pilocytic astrocytoma	1	1
Anaplastic oligodendroglioma	1	0
PXA tumor	1	0
Grade 3 glioma	1	1
Glioma grade		
Grade 2	8	2
Grade 3	4	2
Grade 4	7	3

The interviews identified 21 outcomes, of which 2 were not identified in the registry review or the systematic review of qualitative studies.

### Stage 3

Following removal of 28 duplicates and merging of one outcome, 35 outcomes were reviewed by the research team. The language describing each outcome was refined. The categories used at this stage were: Survival, Resource Use, Adverse Events, Function (changed to “Activities of Daily Living” prior to Delphi), Disease Activity, Health-related Quality of Life, and Symptoms.

### Stage 4

Ninety-six participants were recruited to the Delphi survey between April 4, 2022 and May 8, 2022, during which round 1 was open (Table 2). One participant completed the survey via telephone, all others did so independently online. Round 2 was open from May 13, 2022 to June 12, 2022. Once recruited, monitoring and reminders were used to encourage completion of both survey rounds, resulting in a return rate of 80%.

**Table 2.** Demographic Table for Delphi Participants for Each Round

Delphi survey demographics (both round participants)				
Demographics	Stakeholder			
	Patients	Caregivers	Clinicians/other healthcare professionals	Other
Stakeholder group	34	14	19	Researchers – 4
				Third sector – 4
				Regulator – 2
				Pharmaceutical – 1
Age (range)	22–67	27–70	26–59	25–55
Gender				
Female	16 (47%)	8 (57%)	6 (32%)	7 (70%)
Male	18 (53%)	6 (43%)	13 (68%)	3 (30%)
Ethnicity				
White, British	33 (97%)	14 (100%)	14 (74%)	8 (80%)
Asian/Asian British	1 (3%)	0 (0%)	3 (16%)	0 (0%)
Black/Black British	0 (0%)	0%	1 (5%)	0 (0%)
Other ethnic group	0 (0%)	0%	1 (5%)	1 (10%)
Prefer not to say	0 (0%)	0%	0 (0%)	1 (10%)
Region				
England	27 (79%)	13 (93%)	12 (63%)	6 (60%)
Northern Ireland	1 (3%)	0 (0%)	0 (0%)	0 (0%)
Scotland	4 12%)	1 (7%)	6 (32%)	2 (20%)
Wales	1 (3%)	0 (0%)	1 (5%)	1 (10%)
Prefer not to say	1 (3%)	0 (0%)	0 (0%)	1 (10%)
Participated in a trial				
Yes	8 (24%)			
No	26 (76%)			
Relationship to patient				
Spouse		9 (64%)		
Parent		1 (7%)		
Child		3 (21%)		
Other (Sibling)		1 (7%)		
Self-reported glioma diagnosis				
Astrocytoma	6 (18%)	1 (7%)		
Anaplastic astrocytoma	4 (12%)	1 (7%)		
Glioblastoma	11 (32%)	6 (43%)		
Oligodendroglioma	7 (21%)	2 (14%)		
Anaplastic oligodendroglioma	2 (6%)	0 (0%)		
Diffuse astrocytoma	1 (3%)	0 (0%)		
Neuroectodermal tumor	1 (3%)	0 (0%)		
Optic nerve glioma	0 (0%)	1 (7%)		
Unknown/unclear	2 (6%)	3 (21%)		
Self-reported glioma grade				
Grade 2	12 (35%)	2 (14%)		
Grade 2/3	4 (12%)	1 (7%)		
Grade 3	6 (18%)	0 (0%)		
Grade 3/4	1 (3%)	1 (7%)		
Grade 4	10 (29%)	7 (50%)		
Unknown/unclear	1 (3%)	3 (21%)		
Professional role				
Neurosurgery			10	0
Neuro-oncologist			5	0
Nurse			2	0
Radiographer			1	0
Researcher			0	3
Third sector			0	4
Regulator			0	2
Pharmaceutical			0	1
Other			1	0
Secondary roles				
Caregiver				1
Clinician/other healthcare professional				3
Researcher				8
Policymaker				1
Round 1 demographics—attrition between round 1 to round 2				
Patients	7–1 Glioblastoma, 1 oligodendroglioma, 1 PXA tumor, 1 low-grade glioma, 1 diffuse midline glioma, 1 anaplastic oligodendroglioma, 1 diffuse astrocytoma^*^			
Caregivers	7–4 Carers of glioblastoma, 2 carers of oligodendroglioma, 1 unknown/unclear^*^			
Clinicians/researchers	5–3 Neurosurgeons, 1 oncologist, 1 researcher			

^*^Self-reported diagnoses

A total of 35 outcomes were included in round 1 of the Delphi survey. Twenty-six outcome suggestions were added by participants in round 1, 10 of these were presented in round 2 alongside the original outcomes. Following round 2, 22 outcomes met the criteria for automatic inclusion into the COS (outcome rated 7–9 by ≥70% of respondents and 1–3 by ≤15%). All 22 outcomes were from the original outcome longlist of 35 outcomes.

Forty-nine participants used the free-text comment boxes, providing 217 comments. Of these, 5 comments described changing score based on that of others, 28 described doing so based on the patients’ perspective. In total, 76 comments described feeling differently about the outcome in question compared to when they participated in the first round; 14 described that they had previously misunderstood; and 17 comments appeared to be individuals reporting their own symptoms or experience.

### Stage 5

Twenty-two outcomes met the criteria for automatic inclusion in the COS; these were ratified during the consensus meeting within 7-outcome domains based on their ontological categories. A total of 23 outcomes did not meet the threshold for inclusion or exclusion from the COS so were also discussed during the consensus meeting. None of these 23 “no consensus” outcomes were included in the COS. Three of those that had been automatically included following the Delphi were considered not applicable to all adult primary glioma phase III interventional trials, inclusive of supportive care interventions. These 3 outcomes were related to disease progression. Their removal was proposed during the consensus meeting.

The consensus meeting took place on June 28, 2022 and was attended by 13 people (Table 3).

**Table 3.** Table of Demographic Characteristics of Consensus Meeting Participants

Participant stakeholder group	No	Representation
Patients	3	3 Glioblastoma
Caregivers	2	1 Glioblastoma 1 Astrocytoma
Healthcare professional	5	1 Consultant neurosurgeon 1 Neurosurgeon and researcher 1 Neurosurgery and neuro-oncology PhD candidate 1 Manager of neuro-oncology network 1 Consultant psychiatrist
Researcher	1	1 Clinical researcher
Third sector	1	1 Third sector
Regulator	1	1 Regulator
Policymaker	1*	See healthcare professionals—secondary role of working in “Health Policy” *Duplicate role, not included in final number count
Total	13	

### Final COS

The final COS consists of 7 outcome domains, encompassing 19 outcomes, and was confirmed through 2 rounds of voting. The individual outcomes within each domain, and their definitions, are described in Table 4.

**Table 4.** Final Core Outcome Set, Number of Voting Rounds and Scores

Outcome domains, outcomes, and definitions	Final voting results (consensus meeting)	
	Retain	Remove
Survival Overall survival In a clinical trial, the time to a person’s death from any cause, starting from the time they joined the trial. Survival rate In a clinical trial, the time from when a person starts a trial, to when their disease worsens or they die. Survival without neurocognitive deterioration The time from when a person starts a clinical trial to when their neurocognitive symptoms (eg, memory) become worse or they die due to any cause, whichever occurs first.	75%	25%
Adverse events f adverse events How often a person experiences any unfavorable, unexpected symptoms or signs that may be related to the treatment, including neurological adverse events (eg, seizure activity), in a given time period. Severity of adverse events The severity of any unfavorable, unexpected symptoms or signs a person experiences that may be related to the treatment, including neurological adverse events (eg, seizure activity). Interference of adverse events How unfavorable, unexpected symptoms or signs a person experiences, interferes with their daily activities. These may be related to the treatment, including neurological adverse events (eg, seizure activity). Overall tolerability The degree to which adverse events (symptoms or signs) of the intervention (eg, chemotherapy or peer support) can be tolerated by a person, overall. Evaluation of late adverse events The assessment of unfavorable, unexpected late symptoms or signs of an intervention (eg, chemotherapy or peer support), experienced by a person after the intervention period has finished.	83%	17%
Activities of daily living Activities of daily living—basic A person’s daily functioning including feeding, personal toileting, bathing, dressing and undressing, getting on and off a toilet, controlling bladder, controlling bowel, moving from wheelchair to bed and returning, walking on level surface (or propelling a wheelchair if unable to walk), and ascending and descending stairs. Activities of daily living—instrumental A person’s ability to undertake activities which allow them to live independently and participate in the community (including driving/transportation, work, shopping, cooking meals, participating in social activities). Performance status A measurement of a person’s overall function, including mobility, self-care and work.	92%	8%
Health-related quality of life Health-related quality of life A person’s assessment how their physical, emotional, social, or other types of well-being are affected by glioma or its treatment.	92%	8%
Seizure activity Seizure activity An overall measure of how often a person experiences seizures and their severity.	83%	17%
Neurocognitive function Neurocognitive function An overall assessment of a person’s neurocognitive function. Higher executive function A person’s ability to plan, execute, and monitor their goals. Memory A person’s ability to register and store information, and retrieve it as needed. Dysphasia A person’s experience of difficulty in comprehending or expressing language in its written or spoken form, sometimes described as aphasia.	83%	17%
Physical function Hemiparesis A person’s experience of difficulty or inability to intentionally move parts of the body or to coordinate movements. Vision A person’s experience of partial and/or double vision.	92%	8%

#### Identification of patient-reportable outcomes.

Use of 6 PRO measures (PROMs)^41–46^ were reported by trials in the Stage 1 review, all reported to capture HRQOL aspects. Cross-referencing PROM items with COS subdomains identified overlap with 5 of the 7 core outcomes (adverse events, activities of daily living, seizure activity, neurocognitive function, physical function) (Table 5).

**Table 5.** Trial Registry Review Patient-Reported Outcome Measures Mapped to Final Core Outcome Set Domains

Core outcome set domains	Survival	Adverse events	Activities of daily living	Health-related quality of life	Seizure activity	Cognitive function	Physical function
Registry Review—patient-reported outcome measures (PROM)	-			EQ-5D-5L EORTC QLQ-BN20 EORTC QLQ-C30 MDASI-BT FACT-Br LASA (Linear analog self-­assessment)			
Registry review—quality of life PROM overlap with COS domains	–	FACT-Br –Overall tolerability: GP5 [definition of GP5 below—encompassing frequency, severity and interference of adverse events]	FACT-Br –Activities of daily living (ADL) (instrumental): physical, social, and functional well-being—work, enjoying life, usual activities; additional concerns (driving, independence, basic activities) LASA (writing, mobility, self-care, physical activity, recreation, social life, housework, family relations MDASI-BT ADL (basic and instrumental): B: general activity, walking I: work, enjoyment of life EORTC QLQ-C30 ADL (basic and instrumental) B: carrying shopping, walking, eating, dressing, washing, using toilet; I: work, hobbies, leisure time activities, social and family interference due to symptoms EQ-5D-5L ADL (basic and instrumental)		FACT-Br –Seizure activity: Br2, Br5 MDASI-BT –Seizures EORTC QLQ-BN20 –Seizure (“did you have seizures”)	FACT-Br –Memory Br3 Dysphasia Br8, Br9 –Cognitive and executive function Br1, Br11, Br13, Br15, Br16, Br17 LASA –Speech MDASI-BT –Memory: difficulty remembering –Dysphasia (difficulty speaking) –Cognitive function, executive function –Difficulty understanding, difficulty concentrating EORTC QLQ-BN20 –Dysphasia EORTC QLQ-C30 –Memory –Cognitive function (concentrating, reading, watching TV)	FACT-Br –Hemiparesis: Br19, 20, 21 –Vision: Br6 LASA –Mobility MDASI-BT –Hemiparesis (weakness on one side) –Vision (problems with vision) EORTC QLQ-BN20 –Vision –Hemiparesis

#### Deviations from protocol.

Third sector organizations were recruited to the Delphi survey and consensus meeting as PPI team members identified that this perspective was missing. Due to the large number of outcomes not reaching agreement following the Delphi survey, the study team proposed that only outcomes receiving a rating of 7–9 by ≥60% of respondents were presented to the participants for consideration for inclusion in the COS. A large number of outcomes were automatically included in the COS, and the outcomes were grouped into broad outcome domains based on their original categories.

## Conclusions

This research resulted in a COS comprising all outcome types, recommended for use in adult primary glioma phase III interventional trials, including supportive care. It drew on a comprehensive range of sources, including original qualitative data, to ensure all outcomes prioritized by glioma patients and other key stakeholders were included. Thus, this COS could facilitate collection and reporting of patient-centered trial outcomes representing stakeholder interests, and identifies those that are patient-reportable to integrate further the patient perspective.

The final COS consists of 19 outcomes in 7 outcome domains: survival, adverse events, activities of daily living, health-related quality of life, seizure activity, neurocognitive function, and physical function. Given the overlap with FDA-recommended core PROs (disease-related symptoms, symptomatic adverse events, overall side-effects, physical function, and role function),^16^ this COS could be considered for use in registrational trials after further validation.

Progression-free survival was not included in the final COS. As this COS was developed for all interventional trials, to include supportive care interventions which may impact on survival or seizure-free survival but not on disease progression, the consensus at the final stage was to exclude disease-focused outcomes. Despite this, we acknowledge that disease-focused outcomes are appropriate in other glioma trials, and the application of a COS does not preclude additional measures in individual trials.

Subdomains of 6/7 outcomes included in the final COS can be patient-reported following mapping with PROMs identified from the registry review. This initial mapping was undertaken to identify the potential for patient reporting, rather than a conclusive exercise on which measures to apply, and the implementation of this COS will be facilitated by ongoing research to align relevant outcomes to appropriate measurement tools with respect to several psychometric properties. For example, FDA cites the PRO Common Terminology Criteria for Adverse Events (PRO-CTCAE) to capture symptomatic adverse events (AEs).^16^ This is important given underreporting of symptomatic AEs by clinicians.^19,47^ In addition, the FACT-Br, identified in the registry review, contains an item (FACT GP5) increasingly cited by regulators for use as a single item measure of tolerability linked with the ability or desire of the patient to adhere to the dose or intensity of therapy,^7^ corresponding to the “overall tolerability” subdomain in the COS.

This COS forms part of an international effort to unify and improve practice in neuro-oncology and promote patient-centricity. The Response Assessment in Neuro-Oncology Patient Reported Outcomes (RANO-PRO) working group with colleagues from the Fast Track Core Assessment Group (COA) Group has identified patient-reported core symptoms and functions for high-grade glioma^17^ and the PRO measures already used in brain tumor studies.^14^ The COS developed in this study addressed all outcome types and the wider spectrum of glioma. Nonetheless, alignment of constructs in areas such as cognition, seizures, symptomatic adverse events, and physical/role functioning are identified. A notable exception is the symptom of pain, identified as a construct in the RANO collaborative report with a subsequent analysis demonstrating worsening of this symptom construct with disease progression.^48^ This was not prioritized by any of the stakeholder groups in the COBra study, being excluded by all stakeholder groups during the Delphi phase. Although qualitative interview and Delphi participants were largely based in the UK, the methodological approach used and alignment of the final COS with international outcome standardization in cancer and glioma support its contribution to the ongoing development of an internationally applicable COS. Ongoing collaboration with the RANO-PRO working group and COS development in meningioma^49^ will allow for continued standardization of outcomes and terminology.

### Strengths and Limitations

The study had methodological strengths and weaknesses. Including a diverse data sources ensured identification of wide range of outcomes, including collecting original qualitative data to integrate the patient and caregiver perspective. Trial registry review is increasingly used^50^ and minimizes reliance on often incomplete outcome reporting in glioma trial publications.^51^ There are limitations to this approach—inconsistent registry use globally, questionable completeness and specificity, and outdated entries. However, the quality of registration is improving,^52^ and trial registration is associated with subsequent use and publication of the same outcomes defined in their protocols as in their published reports.^53^

The flexible and responsive recruitment strategy and monitoring enabled through recruitment via charities and social media allowed purposive sampling and participation across the spectrum of glioma. Balanced inclusion of individuals with a range of diagnoses was critical for meeting the study aim to finalize a COS representative of all gliomas, realized through accessible, person-centered study design. However, the recruitment strategy relied on internet-based methods which are increasingly recognized to limit participation from particular groups,^54^ and we were unable to verify participants’ self-reported diagnoses. Recruiting those identifying with ethnicities besides White British was not successful. Data protection standards restricted our efforts to sample on this basis and support groups we identified to promote the study to individuals from specific ethnic backgrounds worked with people with a range of cancers rather than glioma specifically. This has implications for generalizability and further validation with particular groups of interest should be explored where this can be justified, including through additional qualitative exercises with specific groups. While underrepresentation including in terms of ethnic minority status in cancer research is well documented,^55^ evidence suggests that there is limited disproportionate burden of glioma in terms of incidence or survival on this basis,^56^ potentially mediating this limitation. However, future research would benefit from investing resource in anticipating and addressing barriers to participation that limit the generalizability of our findings to a diverse UK population.^57^ We experienced difficulty recruiting pharmaceutical representatives and those from regulatory bodies to the Delphi, despite representation of these within study team contacts. This may be due to small numbers of individuals or reluctance to participate due to perceived impartiality. This may limit whether the COS represents their views and priorities, potential future uptake, and buy-in. However, there is alignment between our COS and core PRO domains favored by the FDA and other regulators.

Free text analyses enabled insight into participants’ experience within the Delphi. Instances where participants described changing their rating based on the views of other stakeholder groups evidences the Delphi consensus-generating process. The outcomes language and definitions and the Delphi survey were codeveloped and piloted with PPI team members and the public to ensure clarity and suit functional requirements of those with glioma.^58^ However, some free text comments show that participants may have misunderstood the purpose of the Delphi and the outcome prioritization process, although interpretation is limited by the brief text provided and being unable to follow-up with them. Further efforts are required to promote accessibility of COS development for members of the public.^59^

## Supplementary Material

vdad096_suppl_Supplementary_Appendix

## References

[1] Goodenberger ML , Jenkins RB. Genetics of adult glioma. Cancer Genet. 2012;205(12):613–621. 23238284 10.1016/j.cancergen.2012.10.009

[2] Ostrom QT , Gittleman H, Fulop J, et al. CBTRUS statistical report: primary brain and central nervous system tumors diagnosed in the United States in 2008-2012. Neuro Oncol. 2015;17(Suppl 4):iv1–iv62. 26511214 10.1093/neuonc/nov189 PMC4623240

[3] Louis DN , Perry A, Reifenberger G, et al. The 2016 World Health Organization classification of tumors of the central nervous system: a summary. Acta Neuropathol. 2016;131(6):803–820. 27157931 10.1007/s00401-016-1545-1

[4] Wood MD , Halfpenny AM, Moore SR. Applications of molecular neuro-oncology - a review of diffuse glioma integrated diagnosis and emerging molecular entities. Diagn Pathol. 2019;14(1):29. 30967140 10.1186/s13000-019-0802-8 PMC6457044

[5] Fallowfield L , Nadler E, Gilloteau I, et al. Quality of survival: a new concept framework to assess the quality of prolonged life in cancer. Exp Rev Qual Life Cancer Care. 2017;2(4):225–232.

[6] Coomans MB , Dirven L, Aaronson N, et al. Factors associated with health-related quality of life (HRQoL) deterioration in glioma patients during the progression-free survival period. Neuro-Oncology. 2022;24(12):2159–2169. 35404443 10.1093/neuonc/noac097 PMC9713503

[7] Basch EC , Hudgens S, Jones, L et al. *Broadening the Definition of Tolerability in Cancer Clinical Trials to Better Measure the Patient Experience*. Friends of Cancer Research; 2015. https://friendsofcancerresearch.org/wp-content/uploads/Comparative-Tolerability-Whitepaper_FINAL.pdf

[8] Agency EM. Appendix 2 to the Guideline on the Evaluation of Anticancer Medicinal Products in Man: The Use of Patient-Reported Outcome (Pro) Measures in Oncology Studies. European Union; 2016. https://www.ema.europa.eu/en/documents/other/appendix-2-guideline-evaluation-anticancer-medicinal-products-man_en.pdf

[9] Stamm TB , Thwaites R, Mosor E, et al. Building a value-based care infrastructure in europe: the health outcomes observatory. NEJM Catalyst. 2021. https://catalyst.nejm.org/doi/full/10.101056/CAT.21.0146.

[10] SISAQOL. *Setting International Standards in Analysing Patient-Reported Outcomes and Quality of Life Endpoints*. SISAQOL IMI; 2022. https://www.sisaqol-imi.org/

[11] Agency MaHpR. *Innovative Licensing and Access Pathway*. Government Digital Service; 2022. https://www.gov.uk/guidance/innovative-licensing-and-access-pathway

[12] Kluetz PG , Slagle A, Papadopoulos EJ, et al. Focusing on core patient-reported outcomes in cancer clinical trials: symptomatic adverse events, physical function, and disease-related symptoms. Clin Cancer Res. 2016;22(7):1553–1558. 26758559 10.1158/1078-0432.CCR-15-2035

[13] Hirsch BR , Califf RM, Cheng SK, et al. Characteristics of oncology clinical trials: insights from a systematic analysis of clinicaltrials.gov. JAMA Intern Med. 2013;173(11):972–979. 23699837 10.1001/jamainternmed.2013.627

[14] Dirven L , Vos ME, Walbert T, et al. Systematic review on the use of patient-reported outcome measures in brain tumor studies: part of the Response Assessment in Neuro-Oncology Patient-Reported Outcome (RANO-PRO) initiative. Neuro-Oncol Pract. 2021;8(8):417–425. 10.1093/nop/npab013 PMC8278354 34277020

[15] Kirkham JJ , Davis K, Altman DG, et al. Core outcome et-STAndards for development: the COS-STAD recommendations. PLoS Med. 2017;14(11):e1002447. 29145404 10.1371/journal.pmed.1002447 PMC5689835

[16] Administration FaD. *Core Patient-Reported Outcomes in Cancer Clinical Trials: Draft Guidance for Industry*. Food and Drug Administration; 2021. https://www.fda.gov/regulatory-information/search-fda-guidance-documents/core-patient-reported-outcomes-cancer-clinical-trials

[17] Armstrong TS , Dirven L, Arons D, et al. Glioma patient-reported outcome assessment in clinical care and research: a response assessment in neuro-oncology collaborative report. Lancet Oncol. 2020;21(2):e97–e103. 32007210 10.1016/S1470-2045(19)30796-X

[18] Pakhomov SV , Jacobsen SJ, Chute CG, Roger VL. Agreement between patient-reported symptoms and their documentation in the medical record. Am J Manag Care. 2008;14(8):530–539. 18690769 PMC2581509

[19] Basch E , Jia X, Heller G, et al. Adverse symptom event reporting by patients vs clinicians: relationships with clinical outcomes. J Natl Cancer Inst. 2009;101(23):1624–1632. 19920223 10.1093/jnci/djp386 PMC2786917

[20] Retzer A , Sivell S, Scott H, et al. Development of a core outcome set and identification of patient-reportable outcomes for primary brain tumour trials: protocol for the COBra study. BMJ Open. 2022;12(9):e057712. 10.1136/bmjopen-2021-057712 PMC9528585 36180121

[21] Kirkham JJ , Gorst S, Altman DG, et al. Core Outcome Set-STAndards for Reporting: the COS-STAR statement. PLoS Med. 2016;13(10):e1002148. 27755541 10.1371/journal.pmed.1002148 PMC5068732

[22] Staniszewska S , Brett J, Simera I, et al. GRIPP2 reporting checklists: tools to improve reporting of patient and public involvement in research. BMJ. 2017;358:j3453. 10.1136/bmj.j3453. 28768629 PMC5539518

[23] Public Involvement in Research Impact Pack. https://www.youtube.com/watch?v=ELVHZPTPpF42022

[24] UK Standards for Public Involvement. National Institute for Health and Care Research; 2019. https://sites.google.com/nihr.ac.uk/pi-standards/home

[25] NIH. clinicaltrials.gov. NIH U.S National Library of Medicine; 2022. https://clinicaltrials.gov/. 10.1080/15360280801989377 28792816

[26] ISRCTN. ISRCTN Registry. Springer Nature; 2022. https://www.isrctn.co/.

[27] Long HA , French DP, Brooks JM. Optimising the value of the critical appraisal skills programme (CASP) tool for quality appraisal in qualitative evidence synthesis. Res Meth Med Health Sci. 2020;1(1):31–42.

[28] Brainstrust: the brain cancer people. Brainstrust. https://brainstrust.org.uk/

[29] The Brain Tumour Charity https://www.thebraintumourcharity.org/

[30] Saunders B , Sim J, Kingstone T, et al. Saturation in qualitative research: exploring its conceptualization and operationalization. Qual Quant. 2018;52(4):1893–1907. 29937585 10.1007/s11135-017-0574-8 PMC5993836

[31] Department of Health. Mental Capacity Act. London: HMSO; 2005.

[32] Lumivero. *Unlock Insights with Qualitative Data Analysis Software*. Lumivero; 2023. https://lumivero.com/products/nvivo/

[33] Braun V , Clarke V. Using thematic analysis in psychology. Qual Res Psychol. 2006;3(2):77–101.

[34] Calvert M , King M, Mercieca-Bebber R, et al. SPIRIT-PRO Extension explanation and elaboration: guidelines for inclusion of patient-reported outcomes in protocols of clinical trials. BMJ Open. 2021;11(6):e045105. 10.1136/bmjopen-2020-045105 PMC8246371 34193486

[35] Baddeley E , Retzer A, Sivell S, et al. The experiences of people with glioma and their caregivers: living with uncertainty and long term consequences (COBra Study). Neuro-Oncology. 2022;24(Supplement_4):iv2–iv2.

[36] Williamson PR , Altman DG, Bagley H, et al. The COMET handbook: version 1.0. Trials. 2017;18(3):280. 28681707 10.1186/s13063-017-1978-4 PMC5499094

[37] Palinkas LA , Horwitz SM, Green CA, et al. Purposeful sampling for qualitative data collection and analysis in mixed method implementation research. Adm Policy Ment Health. 2015;42(5):533–544. 24193818 10.1007/s10488-013-0528-y PMC4012002

[38] DelphiManager. Core Outcome Measures in Effective Trials (COMET) Initiative. https://www.comet-initiative.org/delphimanager/.

[39] ePROVIDE Database. Mapi Trust. https://eprovide.mapi-trust.org/advanced-search.

[40] Retzer A , Baddeley E, Sivell S, Seddon K, Bulbeck H, Nelson A et al. Core outcomes in brain tumour trials - the COBra study review of glioma trial registration data. Neuro-Oncology. 2022;24(suppl_4):i.v.5

[41] Herdman M , Gudex C, Lloyd A, et al. Development and preliminary testing of the new five-level version of EQ-5D (EQ-5D-5L). Qual Life Res. 2011;20(10):1727–1736. 21479777 10.1007/s11136-011-9903-x PMC3220807

[42] Fayers P , Bottomley A. Quality of life research within the EORTC—the EORTC QLQ-C30. Eur J Cancer. 2002;38(Suppl 4):125–133. 10.1016/s0959-8049(01)00448-8 11858978

[43] Taphoorn MJ , Claassens L, Aaronson NK, et al. An international validation study of the EORTC brain cancer module (EORTC QLQ-BN20) for assessing health-related quality of life and symptoms in brain cancer patients. Eur J Cancer. 2010;46(6):1033–1040. 20181476 10.1016/j.ejca.2010.01.012

[44] Armstrong TS , Mendoza T, Gning I, et al. Validation of the M.D. Anderson Symptom Inventory Brain Tumor Module (MDASI-BT). J Neurooncol. 2006;80(1):27–35. 16598415 10.1007/s11060-006-9135-z

[45] Thavarajah N , Bedard G, Zhang L, et al. Psychometric validation of the functional assessment of cancer therapy--brain (FACT-Br) for assessing quality of life in patients with brain metastases. Support Care Cancer. 2014;22(4):1017–1028. 24287508 10.1007/s00520-013-2060-8

[46] Locke DE , Decker PA, Sloan JA, et al. Validation of single-item linear analog scale assessment of quality of life in neuro-oncology patients. J Pain Symptom Manage. 2007;34(6):628–638. 17703910 10.1016/j.jpainsymman.2007.01.016 PMC2732111

[47] Veitch ZW , Shepshelovich D, Gallagher C, et al. Underreporting of symptomatic adverse events in phase I clinical trials. J Natl Cancer Inst. 2021;113(8):980–988. 33616650 10.1093/jnci/djab015 PMC8502480

[48] Vera E , Christ A, Grajkowska E, et al. Relationship between RANO-PRO working group standardised priority constructs and disease progression among malignant glioma patients: a retrospective cohort study. EClinicalMedicine. 2022 Nov 12;55:101718. doi:10.1016/j.eclinm.2022.101718. 36386035 PMC9661442

[49] Millward CP , Armstrong TS, Barrington H, et al. Development of “Core Outcome Sets” for meningioma in clinical studies (The COSMIC Project): protocol for two systematic literature reviews, eDelphi surveys and online consensus meetings. BMJ Open. 2022;12(5):e057384. 10.1136/bmjopen-2021-057384 PMC9086638 35534067

[50] Vodicka E , Kim K, Devine EB, et al. Inclusion of patient-reported outcome measures in registered clinical trials: evidence from ClinicalTrials.gov (2007-2013). Contemp Clin Trials. 2015;43:1–9. 10.101016/j.cct.2015.04.004. 25896116

[51] Garnier L , Charton E, Falcoz A, et al. Quality of patient-reported outcome reporting according to the CONSORT statement in randomized controlled trials with glioblastoma patients. Neuro-Oncol Pract. 2020;8(2):148–159. 10.1093/nop/npaa074 PMC8049443 33898048

[52] Viergever RF , Karam G, Reis A, Ghersi D. The quality of registration of clinical trials: still a problem. PLoS One. 2014;9(1):e84727. 24427293 10.1371/journal.pone.0084727 PMC3888400

[53] Chan A-W , Pello A, Kitchen J, et al. Association of trial registration with reporting of primary outcomes in protocols and publications. JAMA. 2017;318(17):1709–1711. 28892118 10.1001/jama.2017.13001 PMC5818784

[54] van Deursen AJ. Digital inequality during a pandemic: quantitative study of differences in COVID-19-related internet uses and outcomes among the general population. J Med Internet Res. 2020;22(8):e20073. 32750005 10.2196/20073 PMC7446757

[55] Mutale F. Inclusion of racial and ethnic minorities in cancer clinical trials: 30 years after the nih revitalization act, where are we? J Adv Pract Oncol. 2022;13(8):755–757. 36727023 10.6004/jadpro.2022.13.8.2 PMC9881739

[56] Ostrom QT , Cote DJ, Ascha M, Kruchko C, Barnholtz-Sloan JS. Adult glioma incidence and survival by race or ethnicity in the United States from 2000 to 2014. JAMA Oncol. 2018;4(9):1254–1262. 29931168 10.1001/jamaoncol.2018.1789 PMC6143018

[57] Farooqi A , Raghavan, R., Wilson, A et al. *Increasing Participation of Black, Asian and Minority Ethnic (BAME) Groups in Health and Social Care Research* . NIHR; 2018. https://arc-em.nihr.ac.uk/clahrcs-store/increasing-participation-black-asian-and-minority-ethnic-bame-groups-health-and-social

[58] Erharter A , Giesinger J, Kemmler G, et al. Implementation of computer-based quality-of-life monitoring in brain tumor outpatients in routine clinical practice. J Pain Symptom Manage. 2010;39(2):219–229. 20152586 10.1016/j.jpainsymman.2009.06.015

[59] Young B , Bagley H. Including patients in core outcome set development: issues to consider based on three workshops with around 100 international delegates. Research Involvement and Engagement. 2016;2(1):25. 29507761 10.1186/s40900-016-0039-6 PMC5831887

