# Supplementary material for: Development of a core outcome set for use in adult primary glioma phase III interventional trials: A mixed methods study
Source: Neurooncol Adv. 2023 Aug 2;5(1):vdad096. doi: 10.1093/noajnl/vdad096 (PMC10503650; doi:10.1093/noajnl/vdad096)
Supplement: vdad096_suppl_Supplementary_Appendix [file vdad096_suppl_supplementary_appendix.docx]

**N-O-D-23-00023.** **Development of a core outcome set for use in adult primary glioma phase III interventional trials – A mixed methods study**

Appendix 1

**Core Outcome Set-STandards for Reporting: The COS-STAR Statement Checklist**

| **SECTION/TOPIC** | **ITEM No.** | **CHECKLIST ITEM** | **REPORTED ON PAGE NUMBER** |
| --- | --- | --- | --- |
| TITLE/ABSTRACT | | | |
| Title | 1a | Identify in the title that the paper reports the development of a COS | 1 |
| Abstract | 1b | Provide a structured summary | 2-3 |
| INTRODUCTION | | | |
| Background and Objectives | 2a | Describe the background and explain the rationale for developing the COS. | 3-4 |
|  | 2b | Describe the specific objectives with reference to developing a COS. | 4 |
| Scope | 3a | Describe the health condition(s) and population(s) covered by the COS. | 4 |
|  | 3b | Describe the intervention(s) covered by the COS. | 4 |
|  | 3c | Describe the setting(s) in which the COS is to be applied. | 4 |
| METHODS | | | |
| Protocol/Registry Entry | 4 | Indicate where the COS development protocol can be accessed, if available, and/or the study registration details. | 4 |
| Participants | 5 | Describe the rationale for stakeholder groups involved in the COS development process, eligibility criteria for participants from each group, and a description of how the individuals involved were identified. | 5-7 |
| Information Sources | 6a | Describe the information sources used to identify an initial list of outcomes. | 4-6 |
|  | 6b | Describe how outcomes were dropped/combined, with reasons (if applicable). | 6-8 |
| Consensus Process | 7 | Describe how the consensus process was undertaken. | 6-8 |
| Outcome Scoring | 8 | Describe how outcomes were scored and how scores were summarised. | 7 |
| Consensus Definition | 9a | Describe the consensus definition. | 7 |
|  | 9b | Describe the procedure for determining how outcomes were included or excluded from consideration during the consensus process. | 7-9 |
| Ethics and Consent | 10 | Provide a statement regarding the ethics and consent issues for the study. | 4, 5-6 |
| RESULTS | | | |
| Protocol Deviations | 11 | Describe any changes from the protocol (if applicable), with reasons, and describe what impact these changes have on the results. | 10 |
| Participants | 12 | Present data on the number and relevant characteristics of the people involved at all stages of COS development. | Table |
| Outcomes | 13a | List all outcomes considered at the start of the consensus process. |  |
|  | 13b | Describe any new outcomes introduced and any outcomes dropped, with reasons, during the consensus process. |  |
| COS | 14 | List the outcomes in the final COS. | Table |
| DISCUSSION | | | |
| Limitations | 15 | Discuss any limitations in the COS development process. | 11 |
| Conclusions | 16 | Provide an interpretation of the final COS in the context of other evidence, and implications for future research. | 10-11 |
| OTHER INFORMATION | | | |
| Funding | 17 | Describe sources of funding/role of funders. | 1-2 |
| Conflicts of Interest | 18 | Describe any conflicts of interest within the study team and how these were managed. | 2 |

*From: Kirkham JJ, Gorst S, Altman DG, Blazeby JM, Clarke M, Devane D, et al. (2016) Core Outcome Set–STAndards for Reporting: The COS-STAR Statement. PLoS Med 13(10): e1002148. https://doi.org/10.1371/journal.pmed.1002148*

Appendix 2

**Guidance for Reporting Involvement of Patients and the Public: GRIPP2 checklist**

*From: Staniszewska S, Brett J, Simera I, Seers K, Mockford C, Goodlad S et al. GRIPP2 reporting checklists: tools to improve reporting of patient and public involvement in research BMJ 2017; 358 :j3453 doi:10.1136/bmj.j3453*

Appendix 3

**Systematic review of qualitative literature search strategy**

1 exp Glioma/

2 exp Brain Neoplasm/

3 (("Central‐Nervous‐System‐Neoplasms" or malignant) adj glioma*).ti,ab.

4 exp Glioblastoma/

5 exp Neuroectodermal Tumors, Primitive, Peripheral/

6 neuroectodermal tumo?r*.ti,ab.

7 exp Ependymoma/

8 ependymoma*.ti,ab.

9 (brain adj (tumor* or tumour* or neoplas* or malignan* or cancer*)).ti,ab.

10 (glioma* or astrocytoma* or meningioma* or oligodendroglioma* or oligoastrocytoma* or glioblastoma* or GBM* or Glioblastoma multiforme).ti,ab.

11 High grade glioma*.ti,ab.

12 Low grade glioma*.ti,ab.

13 1 or 2 or 3 or 4 or 5 or 6 or 7 or 8 or 9 or 10 or 11 or 12

14 ((("semi-structured" or semistructured or unstructured or informal or "in-depth" or indepth or "face-to-face" or structured or guide) adj3 (interview* or discussion* or questionnaire*)) or (focus group* or qualitative or ethnograph* or fieldwork or "field work" or "key informant")).ti,ab. or interviews as topic/ or focus groups/ or narration/ or Qualitative research/

15 Interview*.ti,ab.

16 (patient reported* or patient-reported* or patient reported outcome* or patient-reported outcome*).ti,ab.

17 (mixed methods or mixed-methods).ti,ab.

18 14 or 15 or 16 or 17

19 13 and 18

20 limit 19 to english language

Appendix 4

**Semi-structured topic guide**

Main questions (questions to be aimed at both patient and caregiver – be sure to check both have had the opportunity to answer to gain perspectives from both sides of the dyad).

1. Can you tell me more about your diagnosis of LGG/HGG?

- What happened and how did they manage to get a diagnosis

2. Can you tell me what you understand about your LGG/HGG?

- What is happening in your body right now, can you explain what LGG/HGG means for you, how is LGG/HGG affecting you

3. What symptoms have you experienced?

- Probe for any differences in symptoms pre and post diagnosis and treament.

4. What treatment have you received? Do you have any treatment planned?

- ‘Treatment’ may include surgery, radiotherapy, chemotherapy

5. What impact do you think the treatment has had on you/your family/friends?

6. What impact do your symptoms have on your day to day life?

- Probe for impact on relationship with others, including caregiver, social activities, work and any other day to day activities

7. Is there anything you do which helps you manage your symptoms?

- This is other than formal treatments and consider formal rehabilitation sessions or more informal activities which may help

8. What care have you received from your doctor or nurse, or any other health care professionals to help you manage your symptoms?

9. In your opinion, what do you think are the most important symptoms to address? What is it that makes them the most important? How will addressing these symptoms improve your day to day life?

10. Thinking of all that we have discussed today, in regard to the treatment you have received, (either current, past or future treatment – dependent on what the patient has experienced) what are the most important aspects following treatment that you were/are hoping will/were addressed, or are there any aspects that you were expecting to be addressed following treatment but were not? (If any, what aspects following treatment improved or did not improve?)

11. Overall, what are your overall experiences of living with LGG/HGG?

- Probe for both positive and negative

12. Is there anything else you would like to say which has not been mentioned during this interview which we need to know?

Appendix 5 - PRISMA FLOW DIAGRAM – Registry Review

Records identified from:

Registers (n = 234)
- Clinical trials.gov (n=151)
- ISRCTN (n=83)

Records removed *before screening*:

Duplicate records removed (n = 39)

Records screened

(n = 195)

Records excluded (n=104):

**Reasons**

Paediatric population (n=32)

Mixed brain tumour trial (n=11)

Phase I or II (n=42)

Non-interventional study (n=17)

Related to intervention side effects but not supportive care (n=2)

Studies included in review

(n = 91)

Appendix 6 - PRISMA FLOW DIAGRAM – Systematic review of qualitative literature

Records identified from:

Databases (n = 8,265)

*Medline (1,490), Embase (3,789), PsycInfo (270), CINAHL (600),
Web of Science (1,627),
Cochrane – trials (466) and SLRs (23)*

Registers (n = 0)

Records removed *before screening*:

Duplicate records removed
(n = 2,763)

Records marked as ineligible by automation tools (n = 0)

Records removed for other reasons (n = 0)

Records screened

(n = 5,502)

Records excluded

(n = 5,466)

Reports sought for retrieval

(n = 36)

Reports not retrieved

(n = 0)

Reports assessed for eligibility

(n = 36)

Reports excluded:

1. Not patient focused experience (e.g. caregiver impacts only) (n = 8)
2. Glioma population indistinguishable (n = 2)
3. Not lived experience (Process specific, information needs, etc) (n = 5)

Studies included in review

(n = 20)

Reports of included studies

(n = 21)
